# Rupture of a Splenic Artery Aneurysm With Gastric Perforation After Pancreaticoduodenectomy: A Case Report

**DOI:** 10.7759/cureus.64450

**Published:** 2024-07-13

**Authors:** Naoki Kawahara, Shu Tanizawa, Koji Morishita

**Affiliations:** 1 Trauma and Acute Critical Care Center, Tokyo Medical and Dental University Hospital, Tokyo, JPN

**Keywords:** postoperative complication, visceral artery aneurysm, artery aneurysm rupture, interventional radiology, splenic artery aneurysm

## Abstract

Splenic artery aneurysm (SAA) is the most common visceral artery aneurysm and can lead to severe outcomes if ruptured. This report presents the case of a 71-year-old female who experienced a sudden and severe gastrointestinal hemorrhage 19 years after undergoing pancreaticoduodenectomy for pancreatic head cancer. The patient arrived at the hospital with signs of shock, and imaging revealed an SAA rupture with associated gastric perforation. Emergency treatment involved endovascular techniques, which stabilized the patient and controlled the bleeding. This case highlights the importance of rapid diagnosis and the effectiveness of endovascular therapy in managing SAA rupture, particularly in patients with complex surgical histories.

## Introduction

Splenic artery aneurysm (SAA) is the third most common intra-abdominal aneurysm and the most common visceral artery aneurysm, accounting for approximately 60% of all visceral artery aneurysms [1,2]. The true prevalence varies widely among reports, ranging from 0.098% in autopsy cases to 0.78% in angiographic examinations and up to 10.4% in autopsy cases of individuals over 60 years of age [3]. Most are asymptomatic, with only 20% of patients experiencing symptoms such as abdominal or chest pain [4]. Females are four times more likely to develop these aneurysms than males. Risk factors include fibromuscular dysplasia, collagen vascular disease, female sex, history of multiple pregnancies, portal hypertension, trauma, atherosclerosis, and hypertension. However, the exact etiology remains unclear [1,4]. Rupture occurs in 2-10% of SAAs [5], and the mortality rate upon rupture is exceedingly high, ranging from 25% to 70% [4,6]. We report a case of SAA rupture with gastric perforation in the chronic phase after pancreaticoduodenectomy for pancreatic head cancer.

## Case presentation

The patient was a 71-year-old female who had undergone pancreaticoduodenectomy using the Imanaga reconstruction method 19 years previously for pancreatic head cancer. The Imanaga method, unlike the Whipple procedure, involves anastomosing the stomach, pancreas, and bile duct sequentially to the small intestine, providing a more physiological reconstruction of the digestive flow [7]. The postoperative course was uneventful. The patient completed 11 courses of adjuvant chemotherapy with gemcitabine and had no recurrence thereafter.

**Figure 1:**
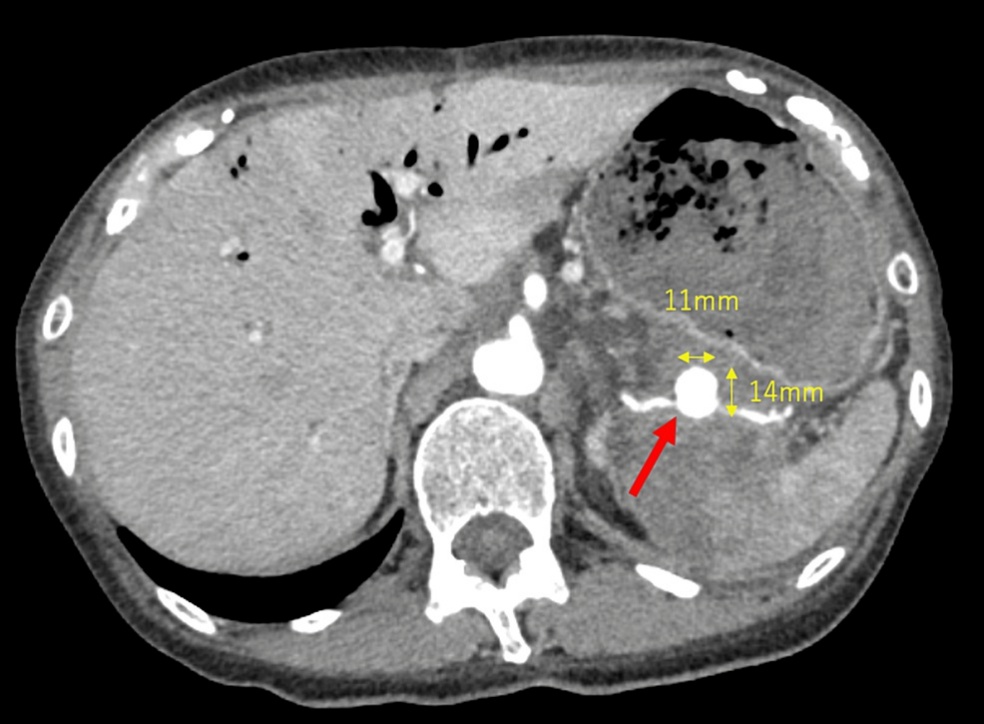
CT findings upon arrival A 14 mm x 11 mm aneurysm with surrounding hematoma in the proximal third of the splenic artery (red arrow) CT: computed tomography

**Figure 2:**
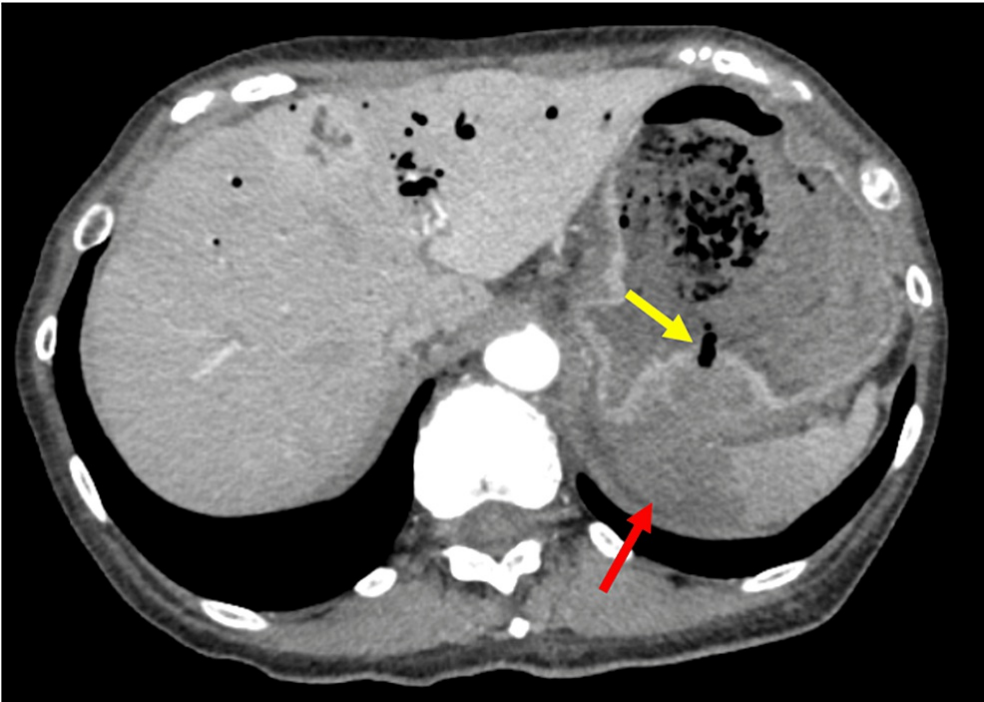
CT findings upon arrival Discontinuity in the posterior gastric wall adjacent to the hematoma around the splenic artery aneurysm (yellow arrow) and evidence of splenic infarction (red arrow) CT: computed tomography

Two weeks before admission, the patient experienced general fatigue, and on the day before admission, she noticed tarry stools. On the day of admission, she had a sudden episode of hematemesis with bright red blood and was urgently transported to our hospital. Upon arrival, she presented with hypotension and tachycardia, which improved after fluid resuscitation by the emergency medical team. Blood tests revealed a hemoglobin level of 4.3 g/dL and an elevated blood urea nitrogen/creatinine ratio. At this point, we suspected upper gastrointestinal bleeding due to gastric or duodenal ulcers. Since the patient's hemodynamics had stabilized with fluid resuscitation, a contrast-enhanced CT scan was performed to identify the source of the bleeding before conducting an emergency upper gastrointestinal endoscopy. CT revealed a 14 mm x 11 mm SAA in the proximal third of the splenic artery with extravasation (Figures 1-2). There was fluid accumulation around the aneurysm, likely to be a hematoma, and a disruption in the continuity of the posterior gastric wall adjacent to the hematoma, indicating a perforation. Infarction was also observed in the spleen.

**Figure 3:**
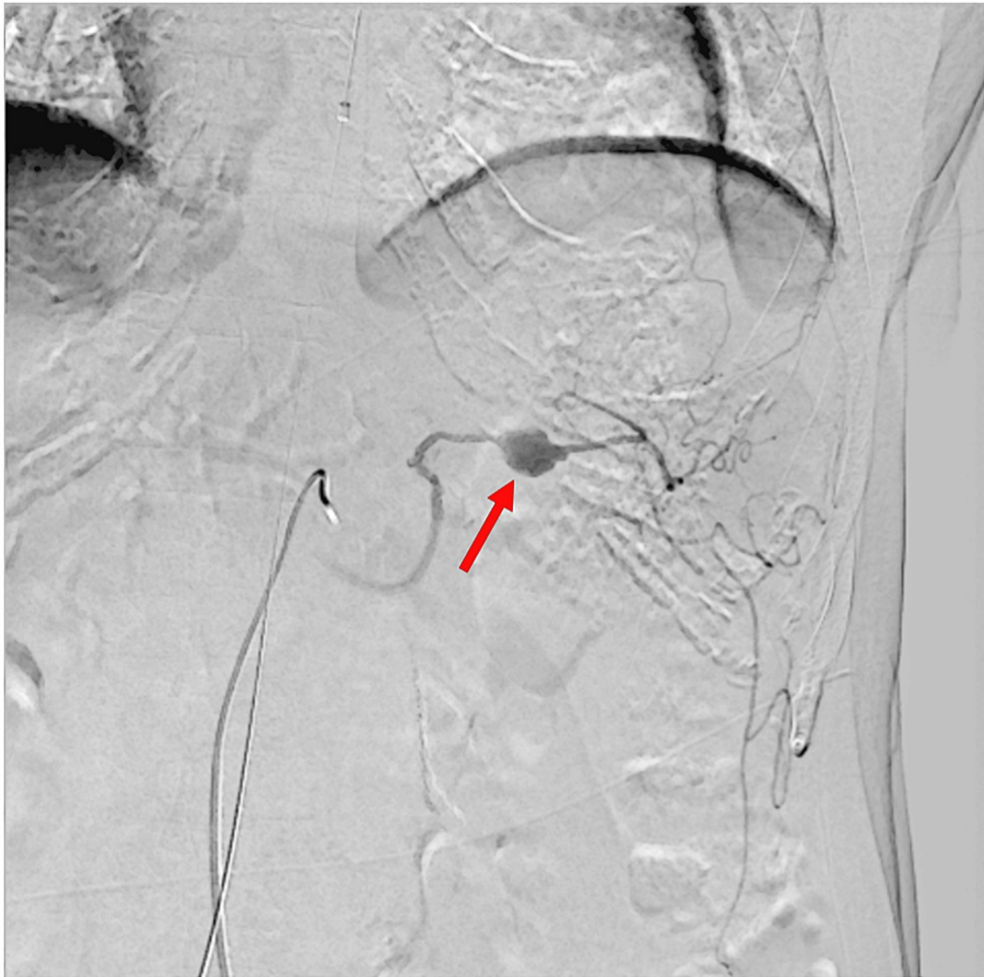
Interventional radiology findings A 14 mm x 11 mm aneurysm in the splenic artery

**Figure 4:**
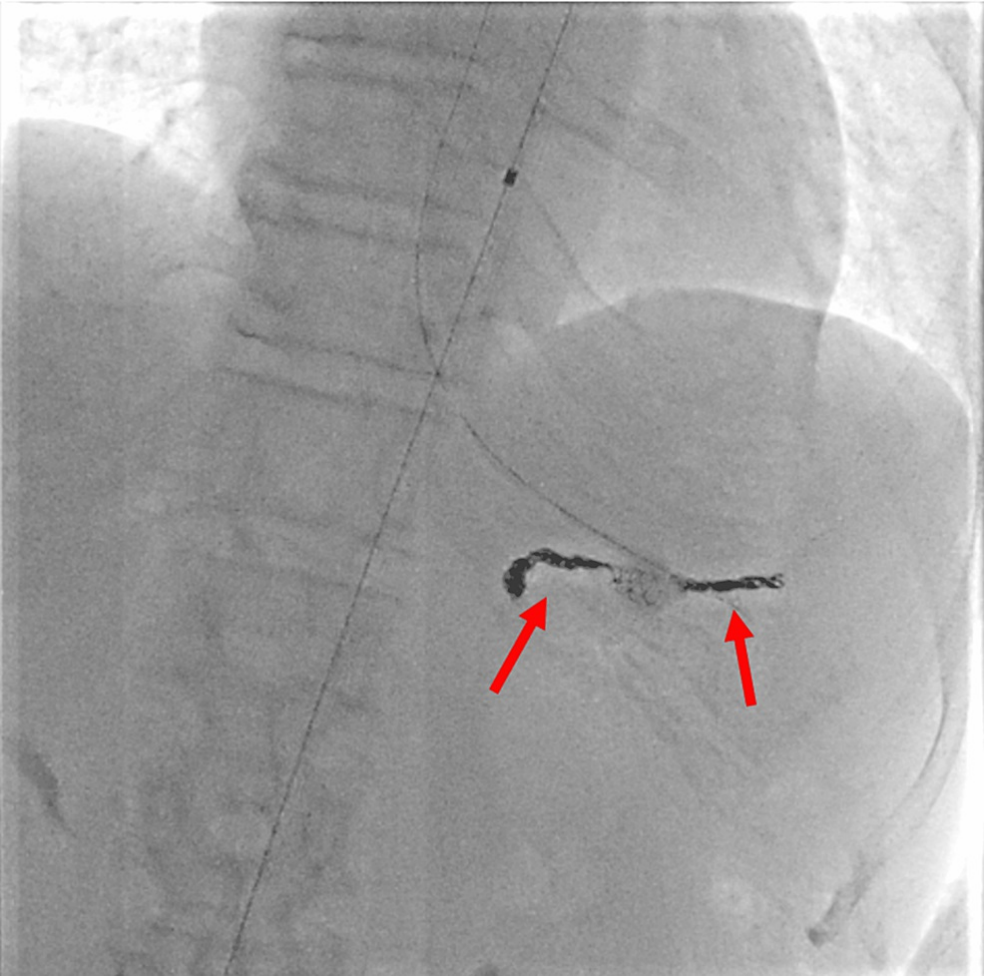
Interventional radiology findings Coil embolization was performed on both the proximal and distal parts of the splenic artery aneurysm.

Based on these findings, a diagnosis of SAA rupture with gastric perforation was made. After a CT scan, the patient experienced massive hematemesis and hypotension. Consequently, we performed endotracheal intubation, and the patient received a transfusion of 8 units of red blood cells and 4 units of fresh frozen plasma. We placed a resuscitative endovascular balloon occlusion of the aorta (REBOA) catheter in aortic zone 1 via the right common femoral artery. Due to an increase in blood pressure, the balloon was kept in standby mode without inflation. Given the stabilized blood pressure, emergency endovascular treatment was performed. Coil embolization was performed on both the proximal and distal parts of the SAA, which controlled bleeding and prevented further hypotension (Figures 3-4).

**Figure 5:**
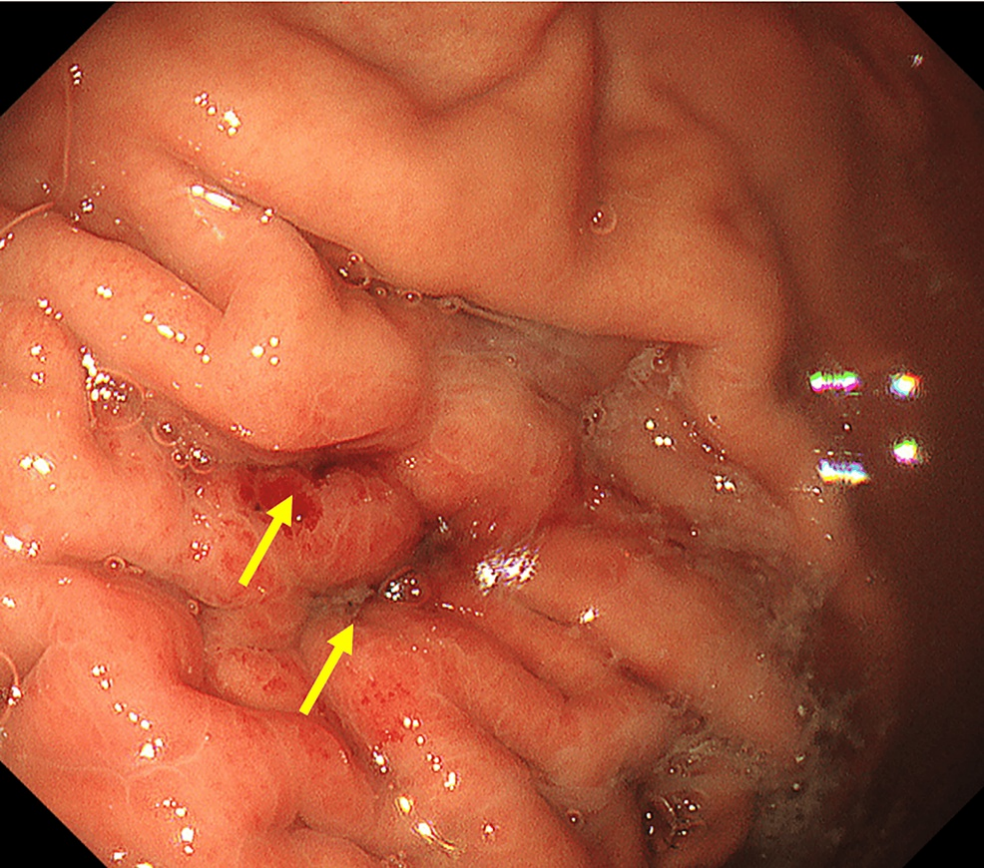
Upper gastrointestinal endoscopy findings on the ninth day of hospitalization An indented area involving the folds was observed in the greater curvature of the upper body of the stomach, which were considered to be the perforation sites.

The patient was then admitted and managed with nil per oral, intravenous fluids, a proton pump inhibitor infusion, antibiotics (ampicillin/sulbactam) to prevent infection of the hematoma, and negative-pressure drainage of the stomach using a nasogastric tube. On the fifth day of hospitalization, the patient was extubated, and the REBOA catheter was removed. A contrast-enhanced CT on the same day showed no new bleeding, reduced fluid collection behind the stomach, or new splenic infarctions. Considering the risk that upper gastrointestinal endoscopy itself could stimulate the aneurysm and exacerbate bleeding and the risk that insufflation could cause gastric contents to flow into the hematoma around the splenic artery aneurysm and lead to infection, we did not perform the endoscopy for several days after admission. On the ninth day of hospitalization, the patient's condition had stabilized, and we believed that these risks had decreased. Therefore, to rule out other conditions, such as gastric ulcer perforation, we performed an upper gastrointestinal endoscopy. The endoscopy revealed a depressed area with folding involvement in the greater curvature of the upper stomach, indicating the perforation sites (Figure 5).

No clear ulcer formation was observed, and given the atypical ulcer site, it was confirmed to be a gastric perforation due to SAA rupture rather than a perforated gastric ulcer. The small size of the perforation and minimal gastric tube drainage allowed for the removal of the nasogastric tube and the resumption of oral intake. There were no signs of increased inflammation after restarting oral intake, and antibiotic treatment was discontinued on the 10th day. The patient was discharged on the 16th day. Two months after discharge, the patient had an uneventful and smooth recovery with no complications.

## Discussion

In this case, the gastric perforation due to a ruptured SAA caused the upper gastrointestinal bleeding. Although their frequency is unknown, SAAs rarely perforate into the stomach, leading to upper gastrointestinal bleeding [8]. As previously mentioned, risk factors for SAA include fibromuscular dysplasia, collagen vascular disease, female sex, history of multiple pregnancies, portal hypertension, trauma, atherosclerosis, and hypertension. However, in this case, the only risk factor identified was being female [1,4]. Despite pancreaticoduodenectomy and chemotherapy, there have been no reports of SAA formation in the chronic phase after pancreaticoduodenectomy or chemotherapy, and the correlation remains unclear. Given the significant postoperative adhesions likely to be present after pancreaticoduodenectomy, the splenic artery might have closely adhered to the surrounding tissues, including the stomach, possibly predisposing the patient to gastric perforation. However, serological tests were not performed; thus, the possibility of underlying conditions such as collagen vascular disease could not be entirely ruled out.

Rare splenic artery pseudoaneurysms can form due to pancreatitis, trauma, iatrogenic causes, surgery, or gastric ulcers [3]. Although this patient had a history of surgery, it had been 19 years, and no pseudoaneurysm was suggested by CT or endovascular treatment. Previous reports recommend intervention for aneurysms larger than 20 mm, with the smallest reported ruptured aneurysm measuring 20 mm [3]. The aneurysm in this case was 14 mm x 11 mm. It is possible that the diameter of the aneurysm decreased due to rupture, but even small aneurysms considered to be at low risk should be evaluated for their potential to rupture.

Treatment options for SAAs include open surgery or endovascular therapy. Surgical options include ligation with or without splenectomy of ruptured SAAs [9]. Although open surgery has a higher success rate, endovascular therapy is associated with fewer complications and lower mortality [3]. Both open surgery and endovascular therapy are options for ruptured SAAs with similar perioperative complication rates; however, endovascular therapy often necessitates further intervention, frequently leading to open surgery [9]. Hemodynamic instability is a risk factor for reintervention with endovascular therapy, suggesting that open surgery should be considered in such cases [9,10]. In this case, the patient was hemodynamically unstable, but because of the anticipated severe adhesions around the splenic artery after pancreaticoduodenectomy, reaching the aneurysm surgically was considered high-risk. Temporary hemodynamic stabilization was achieved with transfusion and REBOA, allowing for a more time-efficient endovascular treatment. Hemostasis was achieved solely with endovascular therapy, avoiding further interventions and allowing early mobilization. Endovascular therapy should be prioritized in cases of anticipated surgical difficulties owing to extensive adhesions.

Intra-abdominal hematomas can become abscessed, necessitating drainage to prevent infection [11,12]. In this case, the risk of an infected hematoma was high because of the continuous hematoma around the gastric perforation. Thus, the patient was managed with nil per oral, negative pressure nasogastric drainage, and prophylactic antibiotics to prevent infection.

## Conclusions

The rupture of SAAs with gastric perforation can cause upper gastrointestinal bleeding. This case demonstrates that endovascular therapy is effective in managing such ruptures, particularly in patients where surgery is challenging due to prior operations and adhesions.

Given the high mortality rate of ruptured SAAs, even small aneurysms may need close monitoring for potential intervention. Recognizing atypical gastrointestinal bleeding and employing a multidisciplinary approach in complex cases is crucial. Further research is needed to improve the understanding and management of SAAs.
